# O07 Educating the public on antimicrobial resistance and stewardship: is there a need to re-direct UK public health campaigns?

**DOI:** 10.1093/jacamr/dlaf118.007

**Published:** 2025-07-14

**Authors:** C Micallef, K Tsipi, S Burridge, C Benney, S Odimayo, S Somerville, N Fleming

**Affiliations:** Cambridgeshire and Peterborough Integrated Care System (ICS), Ely, Cambridgeshire, UK; Health Innovation East, UK; Antibiotic Research, UK; Health Innovation East, UK; Health Innovation East, UK; Antibiotic Research, UK; NHSE East of England, UK

## Abstract

**Background:**

To engage with the public during World Antimicrobial Resistance Awareness Week (WAAW) 2024, several UK local, regional and national organizations, comprising Health Innovation East, Cambridgeshire & Peterborough ICS, Antibiotic Research UK (ANTRUK), and NHSE East of England (EOE), came together and launched an e-platform containing an interactive quiz. This project provided a digital, collaborative and cost-effective way of harnessing the WAAW 2024 theme: Educate, Advocate, Act Now.

**Objectives:**

To engage with a wide audience on antimicrobial resistance (AMR) and antimicrobial stewardship (AMS); provide educational support on common themes around AMR and AMS; and understand the educational needs of the public on AMR and AMS.

**Methods:**

We adapted an evidence-based quiz^1^ involving the Patient and Public Involvement panel from the charity (ANTRUK). A digital platform was built, containing the quiz, comprising a series of 10 true/false statements. Detailed responses, additional information and links to NHS resources were included as feedback when people submitted answers. Each completed quiz generated a donation for ANTRUK as an incentive to complete and submit. The e-platform was beta tested prior to launch, optional demographics were also collected: ethnicity, gender, age-group and post-code. The quiz was launched during a regional WAAW NHSE seminar and through local networks and communication teams from across the region and was live from 18 to 30 November 2024.

**Results:**

The e-quiz was accessed 1876 times with a total of 576 completed responses. It was aimed at and promoted in the East of England but responses were received from across the UK. The two most common incorrect responses marked as True were 'Antibiotic resistance develops as our bodies change and get used to the antibiotics we take so they don't work on us anymore' and Antimicrobial stewardship is an important part of all shipping import processes'. Statistical analysis of number of correct responses for the East of England region (where this quiz was promoted) using a generalized linear mixed effects model (ANOVA and χ^2^) on following demographic data Index of Multiple Deprivation (derived from postcode) age, gender and ethnicity, found that ethnicity was the only statistically significant factor (*P*=0.0184) in number of correct responses.

**Conclusions:**

The most incorrect replies occurred on the questions which focused on how AMR develops and what AMS is. Language used in public health campaigns might not resonate with the public, particularly with those from different ethnicities. More should be done on a national level to inform the public about AMR/AMS using simpler language. These results may be used by local and national policymakers to direct future public educational campaigns on AMR/AMS.

## References

[1] Micallef C, Kildonaviciute K, Castro-Sánchez E et al Patient and public understanding and knowledge of antimicrobial resistance and stewardship in a UK hospital: should public health campaigns change focus? J Antimicrob Chemother 2017; 72: 311–4. 27655854 10.1093/jac/dkw387

